# Supplementary material for: Bioinspired polymeric supramolecular columns as efficient yet controllable artificial light-harvesting platform
Source: Nat Commun. 2024 Jan 30;15:903. doi: 10.1038/s41467-024-45252-9 (PMC10827788; doi:10.1038/s41467-024-45252-9)
Supplement: Supplementary file 1 — Supplementary Information [file 41467_2024_45252_MOESM1_ESM.pdf]

Supplementary Information

Bioinspired polymeric supramolecular columns as efficient yet controllable artificial light-harvesting platform

Bin Mu[1], Xiangnan Hao[1], Xiao Luo[1], Zhongke Yang[1], Huanjun Lu[2] & Wei Tian[1]*

[1]Shanxi Key Laboratory of Macromolecular Science and Technology, Xi'an Key Laboratory of Hybrid Luminescent Materials and Photonic Device, MOE Key Laboratory of Material Physics and Chemistry under Extraordinary Conditions, School of Chemistry and Chemical Engineering, Northwestern Polytechnical University, Xi'an, 710072, China.

[2]Jiangsu Key Laboratory of Micro and Nano Heat Fluid Flow Technology and Energy Application, School of Physical Science and Technology, Suzhou University of Science and Technology, Suzhou, 215009, China.

*E-mail: happytw_3000@nwpu.edu.cn

1. Supplementary Methods

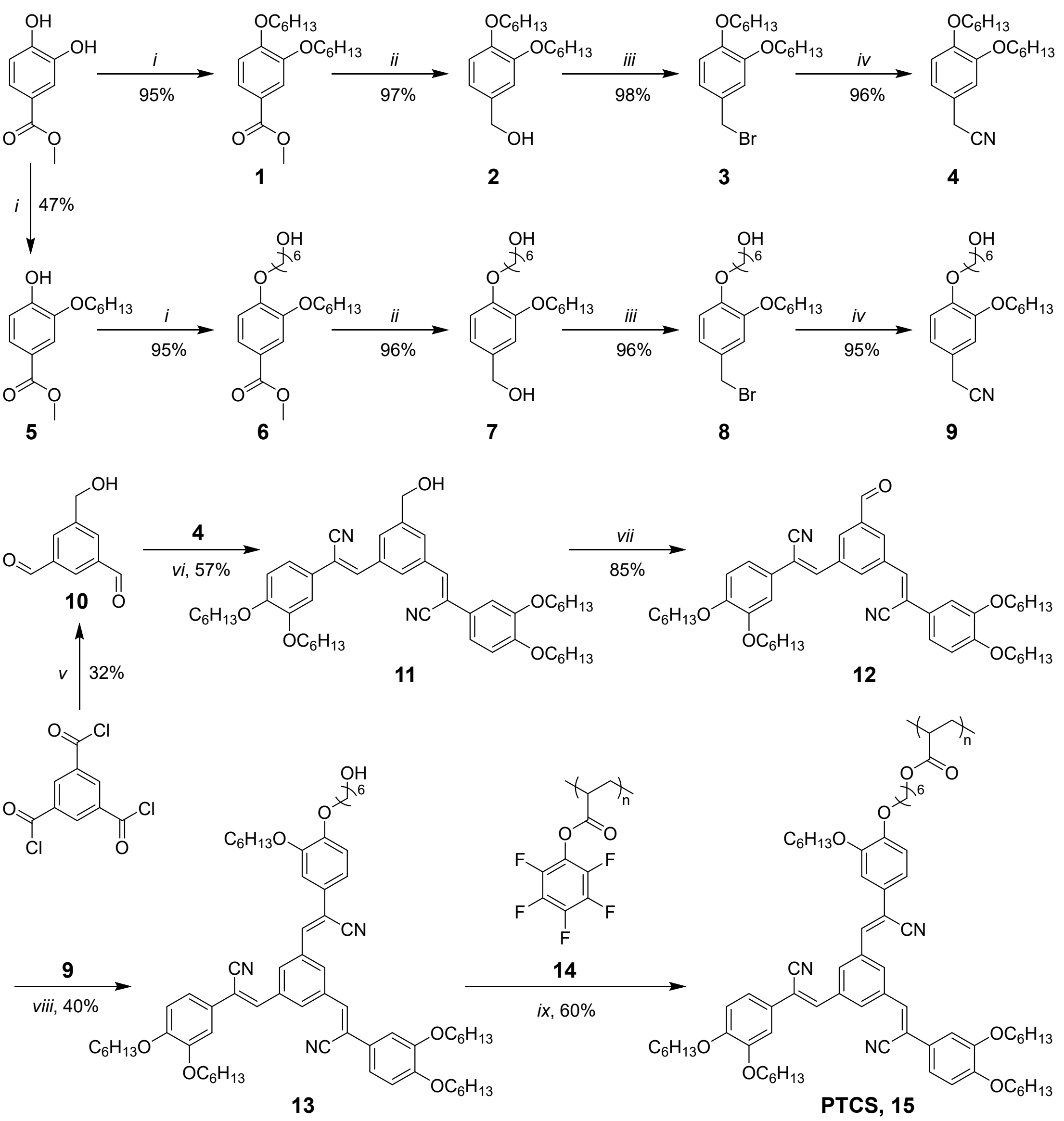

Supplementary Fig. 1 Synthesis of TCS-based LC polymer PTCS. Reagents and conditions: (i) 1-bromohexane, K_2CO_3, tetrabutylammonium iodide, DMF, 85 °C, 12 h; (ii) $LiAlH_4$, THF, 25 °C, 4 h; (iii) PBr_3, dichloromethane, 25 °C, 4 h; (iv) trimethylsilyl cyanide, tetrabutylammonium fluoride, acetonitrile, 25 °C, 4 h; (v) lithium tri-tert-butoxyaluminum hydride, THF, 25 °C, 12 h; (vi) tetrabutylammonium hydroxide, ethanol, 25 °C; (vii) pyridinium chlorochromat, dichloromethane, 25 °C, 12 h; (viii) NaOH, ethanol, 40 °C; (ix) 4-dimethylaminopyridine, DMF, 85 °C, 72 h.

methyl 3,4-bis(hexyloxy)benzoate (1). A mixture of methyl 3,4-dihydroxybenzoate (2.0 g, 11.9 mmol), 1-bromohexane (5.9 g, 35.8 mmol), potassium carbonate (13.4 g, 97.1 mmol) and catalytic amount of tetrabutylammonium iodide in 100 mL DMF was stirred at 85 °C for 12 h. After removal

of the solvent, water was added and extracted with dichloromethane. Finally, the crude product was purified by silica-gel column chromatography using mixed solvents of petroleum ether/dichloromethane (10:1, v/v) as the eluent to give a desired product (3.8 g) in white solid. Yield 95%. ^{1}H NMR (400 MHz, $CDCl_3$): δ (ppm) = 7.64 (dd, 1H, J_1 = 1.7 Hz, J_2 = 8.4 Hz), 7.53 (d, 1H, J = 1.7 Hz), 6.85 (d, 1H, J = 8.4 Hz), 4.04 (m, 4H), 3.88 (s, 3H), 1.83 (m, 4H), 1.54-1.28 (m, 12H), 0.90 (t, 6H, J = 6.7 Hz).

(3,4-bis(hexyloxy)phenyl)methanol (2). To a solution of **1** (3.8 g, 11.3 mmol) in 75 mL THF, lithium aluminium hydride (0.64 g, 16.8 mmol) was added at 0 °C over 2 h and then allowed to stir at room temperature for another 4 h. After adding water to quench the reaction, the mixture was extracted with dichloromethane, dried over anhydrous magnesium sulfate, concentrated and dried under vacuum to give a desired product (3.4 g) in white solid. Yield 97%. ^{1}H NMR (400 MHz, $CDCl_3$): δ (ppm) = 6.92 (s, 1H), 6.85 (s, 2H), 4.60 (s, 2H), 4.00 (m, 4H), 1.82 (m, 4H), 1.52-1.23 (m, 12H), 0.90 (t, 6H, J = 6.8 Hz).

4-(bromomethyl)-1,2-bis(hexyloxy)benzene (3). To a solution of **2** (3.4 g, 11.0 mmol) in 50 mL dichloromethane, a solution of phosphorus tribromide (4.5 g, 16.6 mmol) in 25 mL dichloromethane was added at 0 °C over 2 h and then allowed to stir at room temperature for another 4 h. After adding water to quench the reaction, the mixture was extracted with dichloromethane, dried over anhydrous magnesium sulfate, concentrated and dried under vacuum to give a desired product (4.0 g) in white solid. Yield 98%. ^{1}H NMR (400 MHz, $CDCl_3$): δ (ppm) = 6.92 (m, 2H), 6.80 (m, 1H), 4.48 (s, 2H), 4.00 (m, 4H), 1.82 (m, 4H), 1.55-1.27 (m, 12H), 0.90 (t, 6H, J = 6.9 Hz).

2-(3,4-bis(hexyloxy)phenyl)acetonitrile (4). A solution of **3** (4.0 g, 10.8 mmol), trimethylsilyl cyanide (1.6 g, 16.5 mmol) and tetrabutylammonium fluoride (4.3 g, 16.5 mmol) in 50 mL acetonitrile was stirred at room temperature for 4 h. After removal of the solvent, dichloromethane was added to dissolve the mixture and then washed with water. Finally, the crude product was purified by neutral aluminum oxide column chromatography using dichloromethane as the eluent to give a desired product (3.3 g) in white solid. Yield 96%. ^{1}H NMR (400 MHz, $CDCl_3$): δ (ppm) = 6.83 (m, 3H), 3.98 (m, 4H), 3.67 (s, 2H), 1.81 (m, 4H), 1.53-1.25 (m, 12H), 0.90 (t, 6H, J = 6.9 Hz).

methyl 3-(hexyloxy)-4-hydroxybenzoate (5). A mixture of methyl 3,4-dihydroxybenzoate (2.0 g,

11.9 mmol), 1-bromohexane (1.7 g, 10.2 mmol) and potassium carbonate (3.3 g, 24 mmol) in 100 mL DMF was stirred at 65 °C for 12 h. After removal of the solvent, water was added and extracted with dichloromethane. Finally, the crude product was purified by silica-gel column chromatography using mixed solvents of petroleum ether/dichloromethane (1:1, v/v) as the eluent to give a desired product (1.2 g) in white solid. Yield 47%. ^{1}H NMR (400 MHz, $CDCl_3$): δ (ppm) = 7.59 (m, 2H), 6.85 (d, 1H, *J* = 8.9 Hz), 4.10 (t, 2H, *J* = 6.6 Hz), 3.88 (s, 3H), 1.84 (m, 2H), 1.51-1.20 (m, 6H), 0.92 (t, 3H, *J* = 6.9 Hz).

methyl 3-(hexyloxy)-4-((6-hydroxyhexyl)oxy)benzoate (6). A mixture of **5** (1.2 g, 4.8 mmol), 6-bromo-1-hexanol (1.7 g, 9.4 mmol), potassium carbonate (1.3 g, 9.4 mmol) and catalytic amount of tetrabutylammonium iodide in 50 mL DMF was stirred at 85 °C for 12 h. After removal of the solvent, water was added and extracted with dichloromethane. Finally, the crude product was purified by silica-gel column chromatography using mixed solvents of dichloromethane/ethyl acetate (10:1, v/v) as the eluent to give a desired product (1.6 g) in white solid. Yield 95%. ^{1}H NMR (400 MHz, $CDCl_3$): δ (ppm) = 7.63 (d, 1H, *J* = 8.4 Hz), 7.54 (s, 1H), 6.86 (d, 1H, *J* = 8.4 Hz), 4.05 (t, 4H, *J* = 6.6 Hz), 3.88 (s, 3H), 3.67 (m, 2H), 1.85 (m, 4H), 1.65-1.20 (m, 12H), 0.91 (t, 3H, *J* = 6.8 Hz).

6-(2-(hexyloxy)-4-(hydroxymethyl)phenoxy)hexan-1-ol (7). To a solution of **6** (1.6 g, 4.5 mmol) in 50 mL THF, lithium aluminium hydride (0.26 g, 6.8 mmol) was added at 0 °C over 2 h and then allowed to stir at room temperature for another 4 h. After adding water to quench the reaction, the mixture was extracted with dichloromethane, dried over anhydrous magnesium sulfate, concentrated and dried under vacuum to give a desired product (1.4 g) in white solid. Yield 96%. ^{1}H NMR (400 MHz, $CDCl_3$): δ (ppm) = 6.92 (s, 1H), 6.86 (s, 2H), 4.60 (s, 2H), 4.00 (m, 4H), 3.56 (t, 2H, *J* = 6.4 Hz), 1.82 (m, 4H), 1.65-1.28 (m, 12H), 0.90 (t, 3H, *J* = 6.7 Hz).

6-(4-(bromomethyl)-2-(hexyloxy)phenoxy)hexan-1-ol (8). To a solution of **7** (1.4 g, 4.3 mmol) in 50 mL dichloromethane, a solution of phosphorus tribromide (1.7 g, 6.3 mmol) in 25 mL dichloromethane was added at 0 °C over 2 h and then allowed to stir at room temperature for another 4 h. After adding water to quench the reaction, the mixture was extracted with dichloromethane, dried over anhydrous magnesium sulfate, concentrated and dried under vacuum to give a desired product (1.6 g) in white solid. Yield 96%. ^{1}H NMR (400 MHz, $CDCl_3$): δ (ppm) = 6.92 (d, 1H, *J* = 8.3 Hz), 6.90 (s, 1H), 6.80 (d, 1H, *J* = 8.3 Hz), 4.48 (s, 2H), 4.08 (m, 2H), 3.98 (m, 2H), 1.88-1.66 (m, 6H), 1.57-1.27 (m, 10H),

0.90 (t, 3H, J = 6.9 Hz).

2-(3-(hexyloxy)-4-((6-hydroxyhexyl)oxy)phenyl)acetonitrile (9). A solution of **8** (1.6 g, 4.1 mmol), trimethylsilyl cyanide (0.65 g, 6.6 mmol) and tetrabutylammonium fluoride (1.7 g, 6.6 mmol) in 50 mL acetonitrile was stirred at room temperature for 4 h. After removal of the solvent, dichloromethane was added to dissolve the mixture and then washed with water. Finally, the crude product was purified by neutral aluminum oxide column chromatography using dichloromethane as the eluent to give a desired product (1.3 g) in white solid. Yield 95%. ^{1}H NMR (400 MHz, $CDCl_3$): δ (ppm) = 6.83 (m, 3H), 4.08 (m, 2H), 3.99 (m, 4H), 3.67 (s, 2H), 1.86-1.69 (m, 6H), 1.55-1.20 (m, 10H), 0.90 (t, 3H, J = 5.6 Hz).

1,3-Diformyl-5-hydroxymethylbenzene (10). This compound was synthesized according to the literature procedure[1]. To a solution of lithium tri-tert-butoxyaluminum hydride (8.7 g, 34 mmol) 150 mL THF, a solution of 1,3,5-benzenetricarbonyl trichloride (3.0 g, 11.3 mmol) in 50 mL THF was added dropwise at -78 °C over 2.5 h and then allowed to stir at room temperature for another 12 h. After adding water to quench the reaction, the mixture was extracted with ethyl acetate. Finally, the crude product was purified by silica-gel column chromatography using mixed solvents of petroleum ether/ethyl acetate (1:1, v/v) as the eluent to give a desired product (0.6 g) in white solid. Yield 32%. ^{1}H NMR (400 MHz, $CDCl_3$): δ (ppm) = 10.13 (s, 2H), 8.30 (s, 1H), 8.17 (s, 2H), 4.90 (s, 2H).

(2Z,2'Z)-3,3'-(5-(hydroxymethyl)-1,3-phenylene)bis(2-(3,4-bis(hexyloxy)phenyl)acrylonitrile) (11). To a solution of **10** (0.5 g, 3.0 mmol) and **4** (2.4 g, 7.6 mmol) in 50 mL ethanol, tetrabutyl ammonium hydroxide (40% in methanol, 6 mL, 7.4 mmol) was added and then allowed to stir at room temperature with TLC monitoring. The reaction mixture was poured into water and then extracted with dichloromethane. The crude product was purified by silica-gel column chromatography using mixed solvents of petroleum ether/dichloromethane (1:1, v/v) as the eluent to give a desired product (1.3 g) in light yellow solid. Yield 57%. ^{1}H NMR (400 MHz, $CDCl_3$): δ (ppm) = 8.11 (s, 1H), 7.94 (s, 2H), 7.47 (s, 2H), 7.24 (dd, 2H, J_1 = 2.2 Hz, J_2 = 8.6 Hz), 7.17 (d, 2H, J = 2.2 Hz), 6.92 (d, 2H, J = 8.6 Hz), 4.84 (s, 2H), 4.06 (m, 8H), 1.85 (m, 8H), 1.60-1.20 (m, 24H), 0.91 (t, 12H, J = 6.9Hz).

(2Z,2'Z)-3,3'-(5-formyl-1,3-phenylene)bis(2-(3,4-bis(hexyloxy)phenyl)acrylonitrile) (12). A solution of **11** (1.3 g, 1.7 mmol), celatom (2 g) and pyridinium chlorochromate (0.4 g, 1.9 mmol) in 50

mL dichloromethane was stirred at room temperature for 12 h. After filtration to remove the insoluble residues, the crude product was purified by silica-gel column chromatography using dichloromethane as the eluent to give a desired product (1.1 g) in light yellow solid. Yield 85%. ^{1}H NMR (400 MHz, $CDCl_3$): δ (ppm) = 10.14 (s, 1H), 8.50 (s, 1H), 8.36 (s, 2H), 7.51 (s, 2H), 7.28 (dd, 2H, J_1 = 2.3 Hz, J_2 = 8.4 Hz), 7.19 (d, 2H, J = 2.3 Hz), 6.93 (d, 2H, J = 8.4 Hz), 4.07 (m, 8H), 1.85 (m, 8H), 1.60-1.20 (m, 24H), 0.92 (t, 12H, J = 6.9Hz).

(2Z,2'Z)-3,3'-(5-((Z)-2-cyano-2-(3-(hexyloxy)-4-((6-hydroxyhexyl)oxy)phenyl)vinyl)-1,3-phenylene)bis(2-(3,4-bis(hexyloxy)phenyl)acrylonitrile) (13). To a solution of **12** (1.1 g, 1.4 mmol) and **9** (0.8 g, 2.4 mmol) in 100 mL ethanol, 10 mL saturated solution of sodium hydroxide in ethanol was added and then allowed to stir at 40 °C with TLC monitoring. The reaction mixture was poured into water and then extracted with dichloromethane. The crude product was purified by silica-gel column chromatography using dichloromethane as the eluent to give a desired product (0.6 g) in light yellow solid. Yield 40%. ^{1}H NMR (400 MHz, $CDCl_3$): δ (ppm) = 8.29, 8.27 (s, 3H), 7.50 (s, 3H), 7.29 (d, 3H, J = 8.4 Hz), 7.19 (s, 3H), 6.93 (d, 3H, J = 8.4 Hz), 4.07 (m, 12H), 3.67 (s, 2H), 1.86 (m, 12H), 1.65-1.22 (m, 36H), 0.92 (t, 15H, J = 5.7 Hz). ^{13}C NMR (100 MHz, $CDCl_3$): δ (ppm) = 149.80, 148.41, 148.29, 137.15, 137.11, 134.47, 129.21, 129.10, 125.44, 118.64, 118.56, 116.69, 112.94, 112.89, 112.32, 110.45, 110.35, 68.60, 68.43, 68.21, 61.86, 31.67, 30.58, 30.55, 28.23, 28.11, 24.80, 24.69, 24.66, 24.48, 21.59, 13.00. MALDI-TOF-MS (m/z): $[M + Na]^+$ calcd 1098.69, found 1098.68.

Poly(pentafluorophenyl acrylate) (14). A 50% (w/v) monomer solution in dioxane was used, and the polymerization reaction was carried out under nitrogen at 75 °C for 24 h in a 10 mL pear-shaped Schlenk flask. AIBN was used as the radical initiator, maintaining a molar ratio of AIBN to monomer at 1:50. Before initiation, the polymerization solution underwent three freeze-pump-thaw cycles for degassing. Subsequently, the reaction mixture was purified via silica-gel column chromatography using petroleum ether/DCM (3:1, v/v) as the eluent, affording the polymer product (0.86 g) in a white solid. Yield 86%.

TCS-based LC polymer (PTCS, 15). The trans-esterification was performed by referring to the literature method[2]. **14** (107 mg, 0.45 mmol) was dissolved in 10 mL dry DMF, followed by the addition of **13** (0.5 g, 0.5 mmol) and DMAP (6 mg, 0.05mmol). The mixture was stirred for 72 h at 85 °C. After removing the DMF, the resulting polymers underwent purification through dialysis in DMF (5000) for

eight cycles. This process yielded a polymer product (0.25 g) in a light yellow solid. Yield 53%.

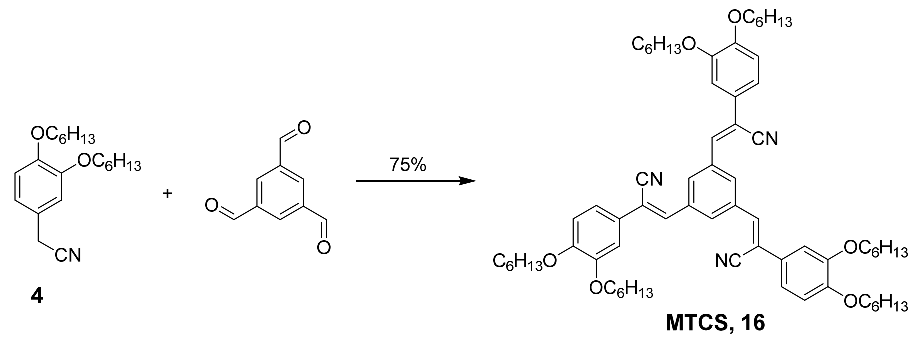

Supplementary Fig. 2 Synthesis of MTCS. Reagents and conditions: tetrabutylammonium hydroxide, ethanol, 25 °C.

(2Z,2'Z,2''Z)-3,3',3''-(benzene-1,3,5-triyl)tris(2-(3,4-bis(hexyloxy)phenyl)acrylonitrile) (MTCS, 16). This control compound was synthesized according to a similar procedure to **11**. Yield 75%. ^{1}H NMR (400 MHz, $CDCl_3$): δ (ppm) = 8.29 (s, 3H), 7.50 (s, 3H), 7.27 (d, 3H, J = 8.4 Hz), 7.19 (s, 3H), 6.93 (d, 3H, J = 8.4 Hz), 4.07 (m, 12H), 1.86 (m, 12H), 1.65-1.22 (m, 36H), 0.92 (t, 18H, J = 6.4 Hz). ^{13}C NMR (100 MHz, $CDCl_3$): δ (ppm) = 149.80, 148.40, 137.08, 134.51, 129.08, 125.44, 118.51, 116.65, 112.94, 112.30, 110.37, 68.60, 68.21, 30.48, 28.16, 24.63, 21.57, 12.96. MALDI-TOF-MS (m/z): M^+ calcd 1059.71, found 1059.66.

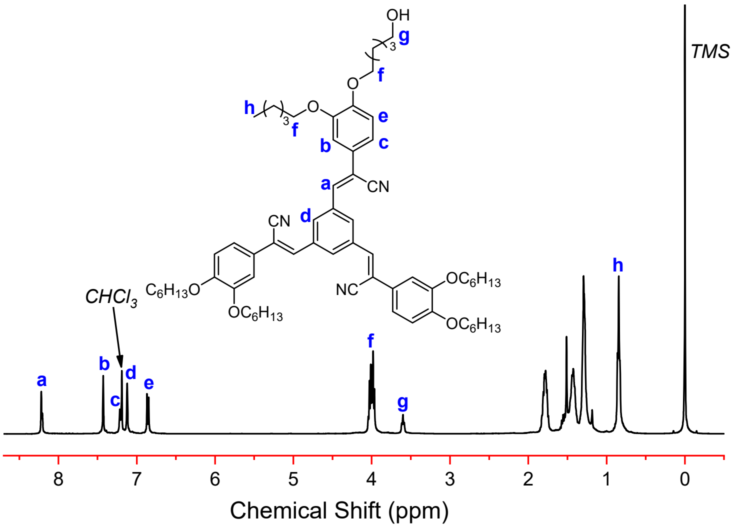

Supplementary Fig. 3 ^{1}H NMR spectrum of monohydroxyl-functionalized TCS monomer **13** in $CDCl_3$.

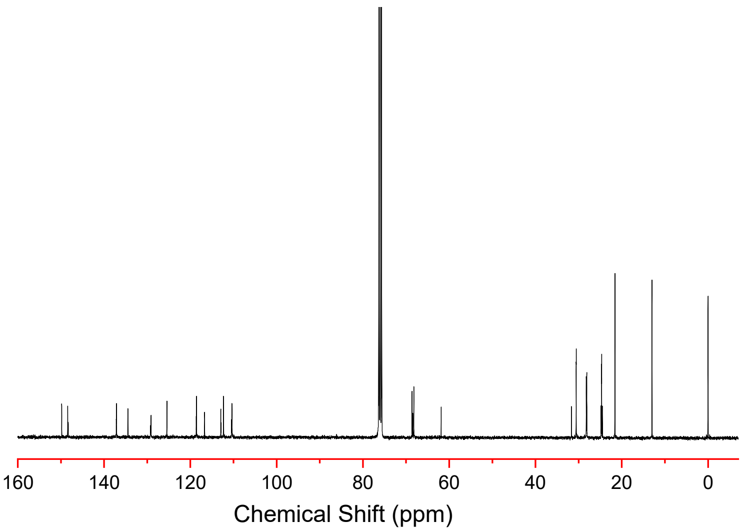

Supplementary Fig. 4 ^{13}C NMR spectrum of monohydroxyl-functionalized TCS monomer **13** in $CDCl_3$.

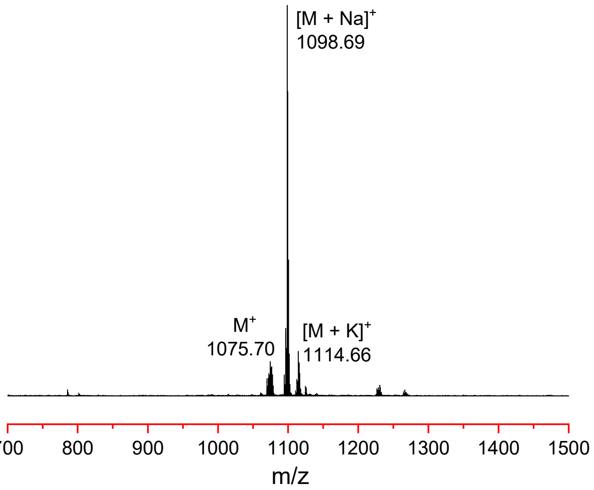

Supplementary Fig. 5 MALDI-TOF-MS spectrum of monohydroxyl-functionalized TCS monomer **13**.

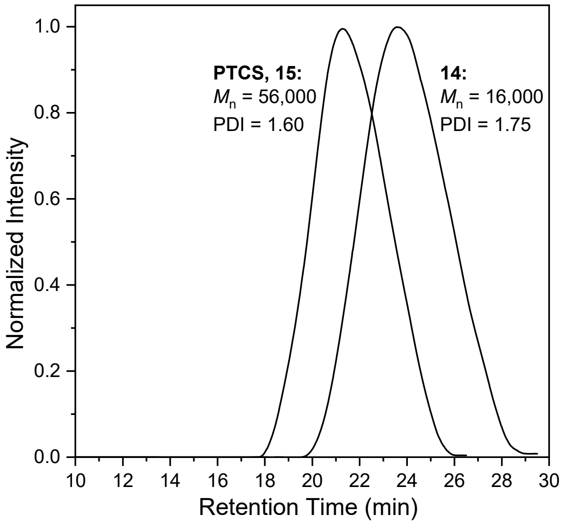

Supplementary Fig. 6 GPC trace comparison of precursor poly(pentafluorophenyl acrylate) (**14**) and the target PTCS (**15**) indicated with calculated number average molecular weight (M_n) and polydispersity (PDI).

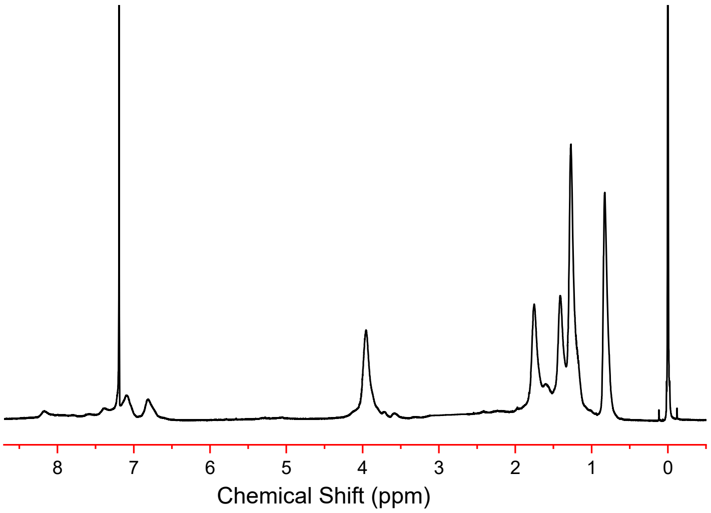

Supplementary Fig. 7 ^{1}H NMR spectrum of PTCS (**15**) in $CDCl_3$ manifesting the successful attachment of TCS mesogens to the polyacrylate.

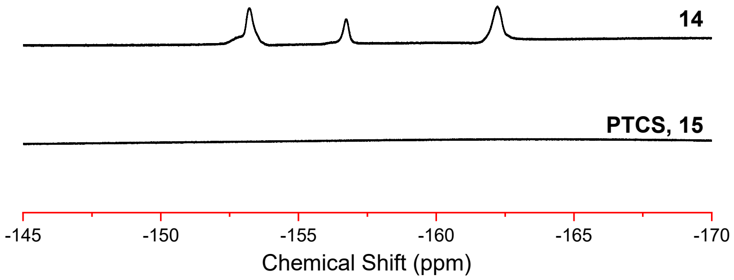

Supplementary Fig. 8 ^{19}F NMR spectra comparison of precursor poly(pentafluorophenyl acrylate) (**14**) and the target PTCS (**15**) in $CDCl_3$. The absence of ^{19}F signal for PTCS confirms the complete transesterification.

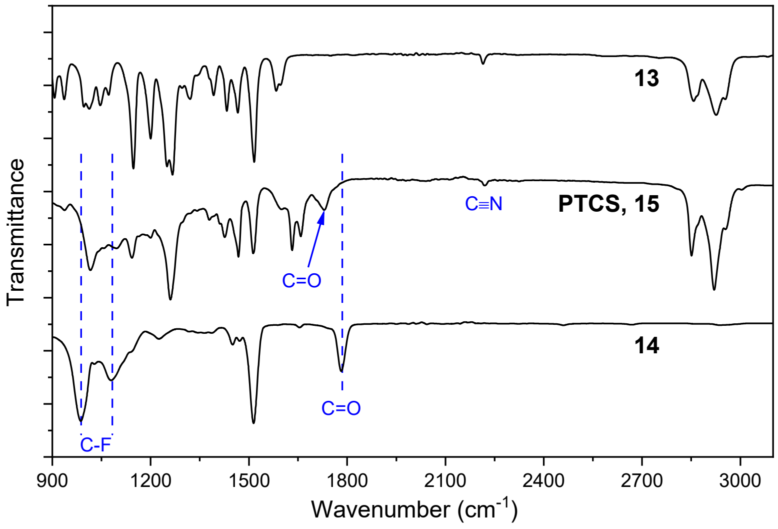

Supplementary Fig. 9 FTIR spectra comparison of precursor monohydroxyl-functionalized TCS monomer (**13**), poly(pentafluorophenyl acrylate) (**14**) and the target PTCS (**15**) for confirming the complete polymer analogous reaction.

2. Supplementary Note 1: Assembly Behaviors

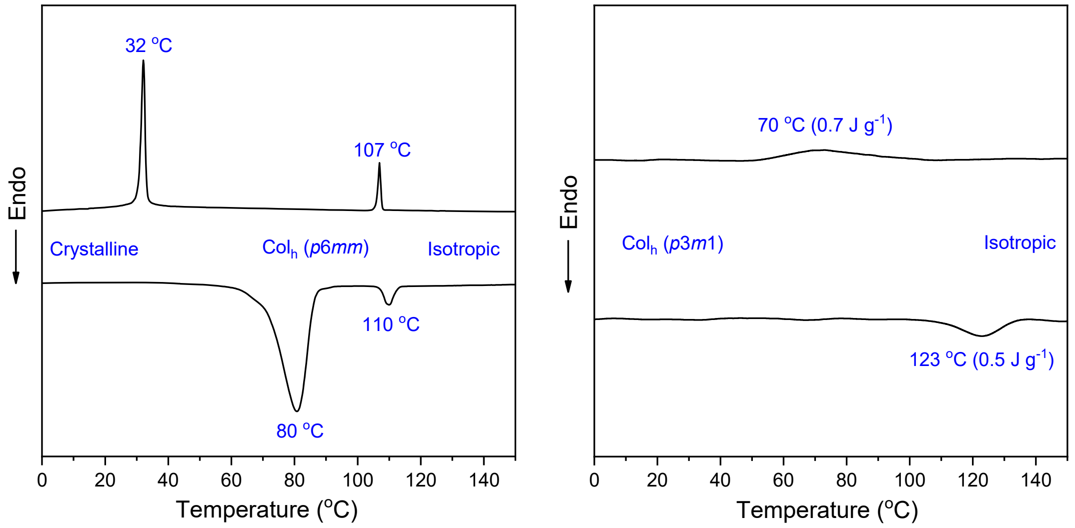

Supplementary Fig. 10 DSC thermograms of (left) MTCS at a scanning rate of 10 °C min^{-1} and (right) PTCS at a scanning rate of 0.5 °C min^{-1}.

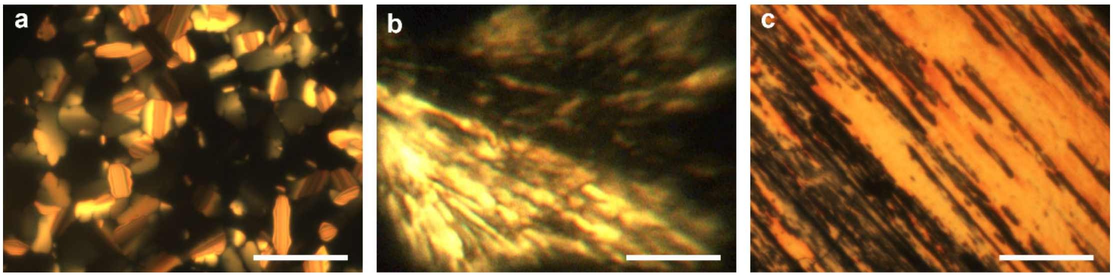

Supplementary Fig. 11 POM images of (a) MTCS at 90 °C after cooling at 10 °C min^{-1}, (b) PTCS at 40 °C after cooling at 1 °C min^{-1}, and (c) PTCS at 40 °C after mechanic shearing. Scale bar is 100 μm.

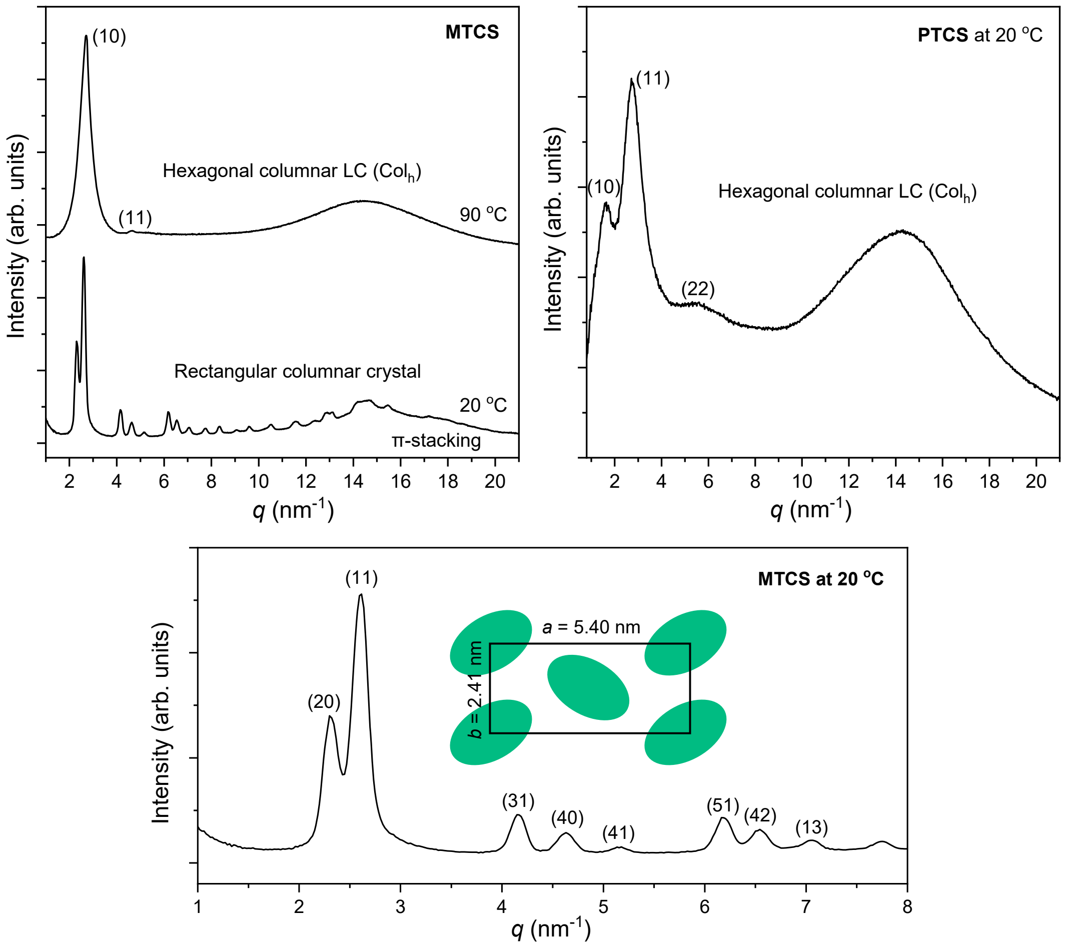

Supplementary Fig. 12 X-ray scattering profiles of MTCS and PTCS at indicated temperatures. MTCS exhibits Col$_h$ LC phase at 90 °C and rectangular columnar crystalline phase (*p*2*gg*, extinction rule: *h*0, *h* = 2*n* + 1; 0*k*, *k* = 2*n* + 1, *n* is an integer) at 20 °C. PTCS exhibits Col$_h$ LC phase at 20 °C.

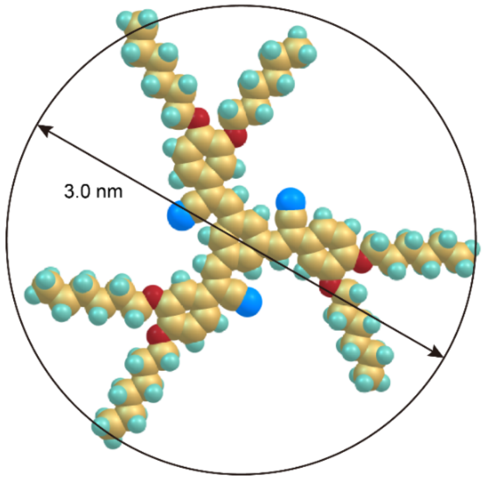

Supplementary Fig. 13 Geometry of MTCS indicated with the measured diameter of the discotic molecule. The X-ray determined diameter 2.68 nm is slightly smaller than the calculated value 3.0 nm due to the alkyl shrinkage and intercolumnar interdigitation, which is necessary to induce columnar mesophase and space filling of the aliphatic chains around the core.

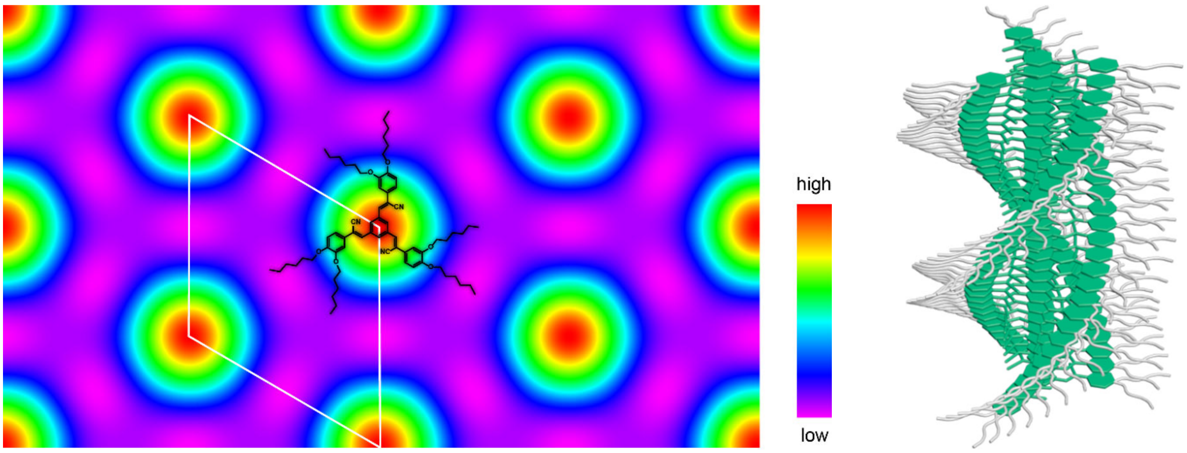

Supplementary Fig. 14 Reconstructed electron density map for MTCS in Col_h (*p6mm*) phase, with representation of molecule overlaid. White parallelogram is the crystallographic unit cell. The conjugated TCS core comprise the areas of highest electron density (red and green), followed by a small rim of ether groups of medium average electron density (blue) and the lowest density moats surrounding by terminal alkyl chains (purple). It is nearly circular in shape owing to orientational averaging by adopting helical stacking manner of the three-armed MTCS molecules.

3. Supplementary Note 2: Light Harvesting Properties

Energy transfer efficiency Φ_{ET}, the fraction of the absorbed energy that is transferred to the acceptor, was calculated based on the fluorescence intensities of the donor in the absence and presence of the acceptor when excited at the maximum absorption of the donor at 365 nm:

$$\Phi_{ET} = 1 - I_{DA} / I_{D}$$

where I_{DA} and I_{D} are the fluorescence intensities arising from the donor (TCS) emission with and without acceptor (NiR), respectively.

The antenna effect (AE) was calculated based on the fluorescence intensities of the acceptor when excited at the maximum absorption of the donor at 365 nm and the maximum absorption of the acceptor at 530 nm:

$$AE = (I_{DA,365} - I_{D,365}) / I_{DA,530}$$

where $I_{DA,365}$ and $I_{DA,530}$ are the fluorescence intensities arising from the acceptor (NiR) when excited at 365 nm and 530 nm, respectively, $I_{D,365}$ is the fluorescence intensity of neat donor (TCS) when excited at 365 nm.

Average number of donor molecules quenched by single acceptor (K_{SV}) was calculated by using

the Stern-Volmer equation:

$$F_0 \, / \, F = 1 + K_{SV} \, [\text{acceptor}]$$

where F_0 and F are the fluorescence intensities arising from the donor (TCS) in the absence and presence of acceptor, respectively, [acceptor] is the concentration of the acceptor. The quenching constant K_{SV} is obtained from the slope of a linear fit to a plot of $F_0 \, / \, F$ versus [acceptor].

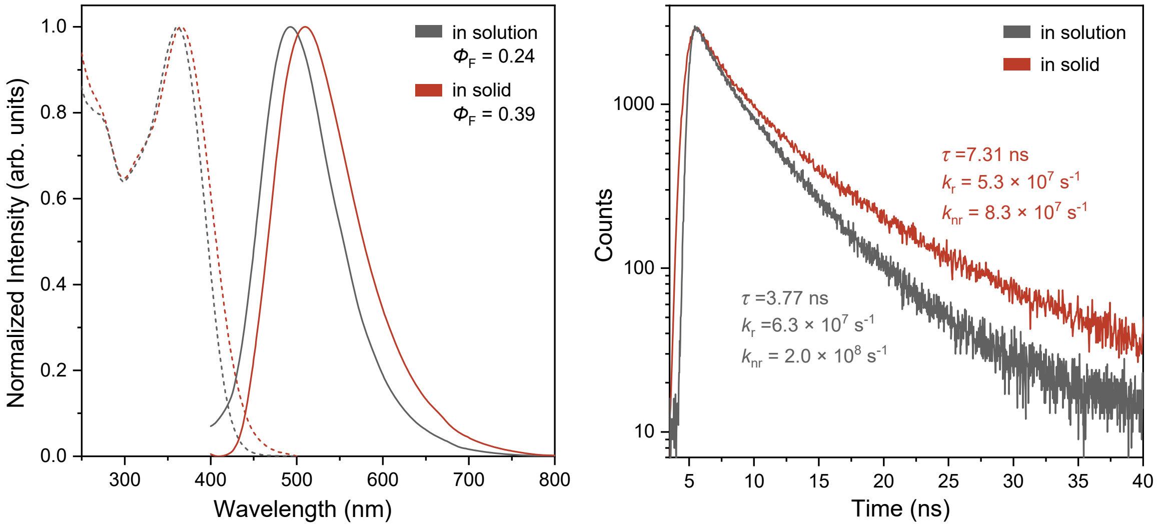

Supplementary Fig. 15 (left) Normalized absorption (dashed line) and emission (solid line) spectra of PTCS in both dichloromethane solution and solid states. The absolute quantum yield (Φ_F) is indicated. (right) Fluorescence decay profiles of PTCS in both dichloromethane solution and solid states. The calculated radiative rate constant (k_r) and nonradiative rate constant (k_{nr}) are indicated.

The PTCS exhibited a larger Φ_F in its solid state compared to its solution state. This indicates an aggregation-enhanced emission characteristic, which can be explained by the reduction of nonradiative decay pathways due to the ordered columnar stacking. Although k_r values are similar, k_{nr} in the solid state is about one third of that in the solution state.

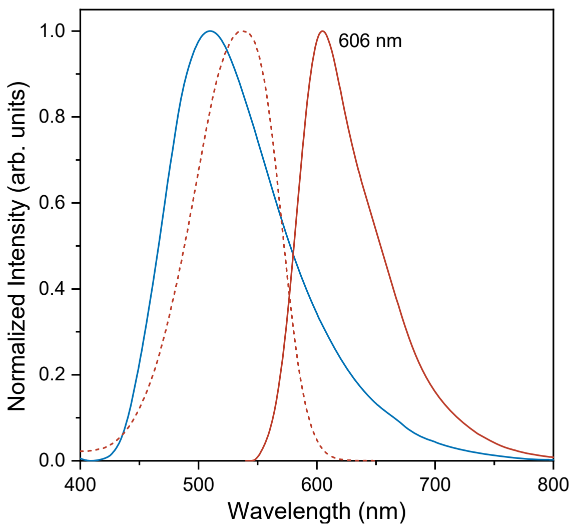

Supplementary Fig. 16 Normalized absorption (dashed line) and fluorescence emission (solid line) spectra of PTCS in the solid state (blue line) or NiR in dichloromethane solution (red line).

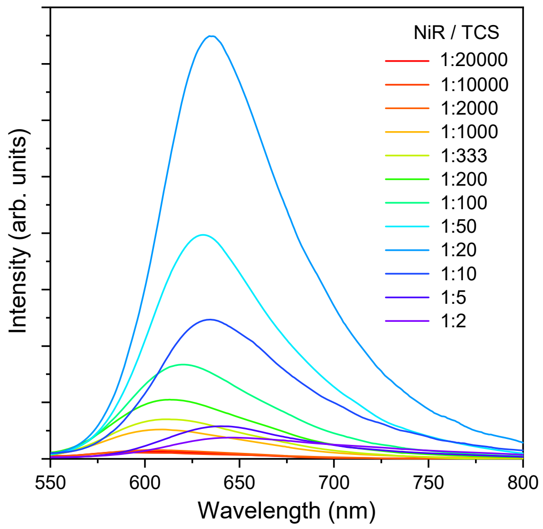

Supplementary Fig. 17 Fluorescence emission spectra of PTCS-NiR with variable ratios of NiR to TCS unit. The excitation wavelength is 530 nm of the maximum absorption of NiR.

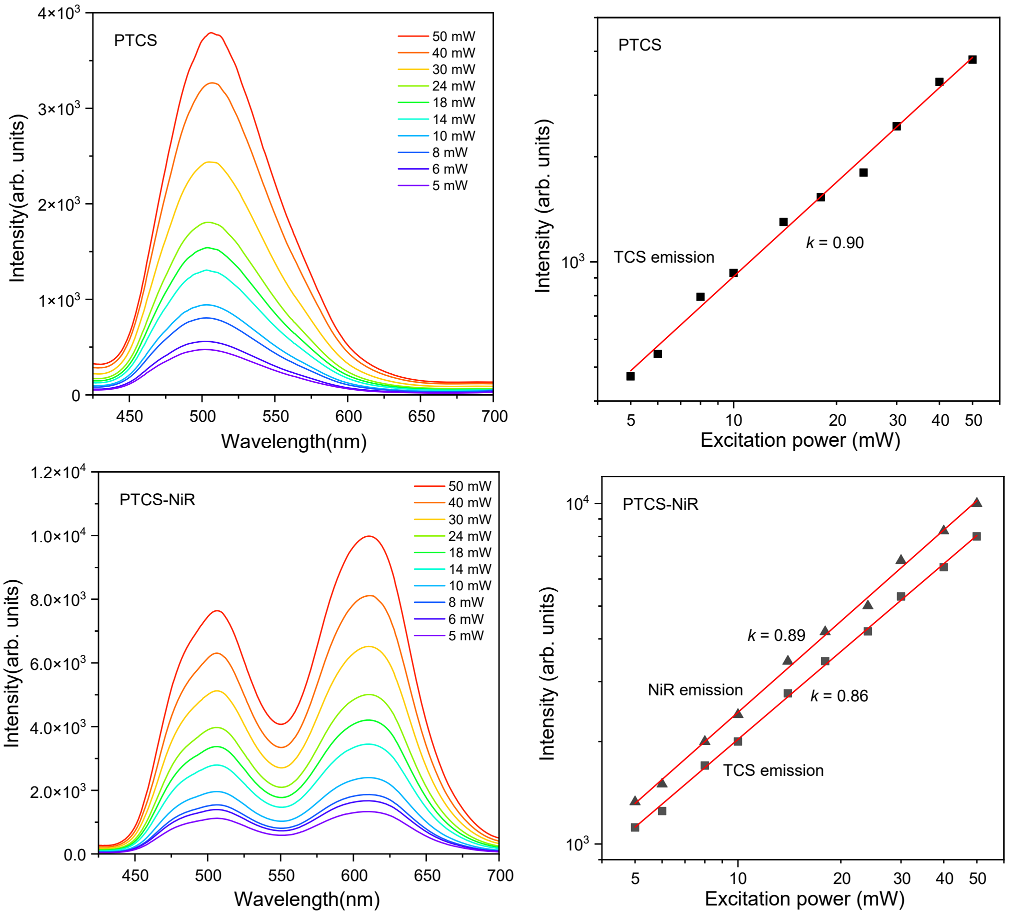

Supplementary Fig. 18 Power-dependent fluorescence spectra and the intensity as a function of excitation power of PTCS and PTCS-NiR with the NiR/TCS ratio of 1:10000.

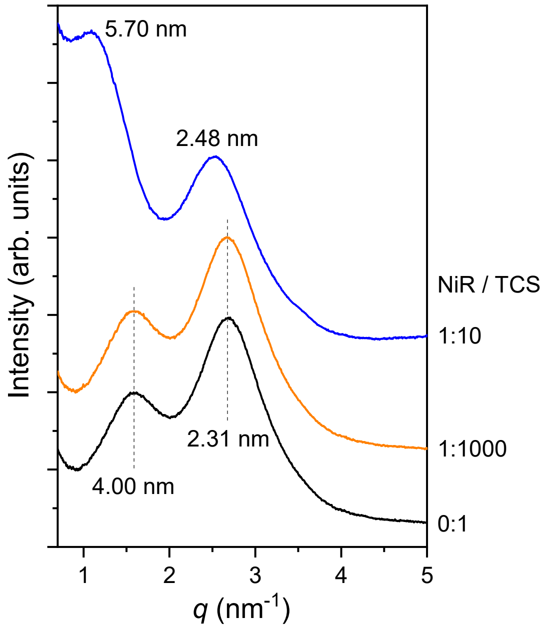

Supplementary Fig. 19 Representative X-ray scattering profiles of PTCS-NiR with different ratios of NiR to TCS unit. The neat PTCS forms Col_h phase, showing two strong scattering peaks with a *d*-ratio of $1:1/\sqrt{3}$. After intercalation of NiR, the Col_h can be largely maintained at small ratios such as 1:1000, but the two-dimensional hexagonal symmetry may be destroyed with a higher ratio such as 1:10.

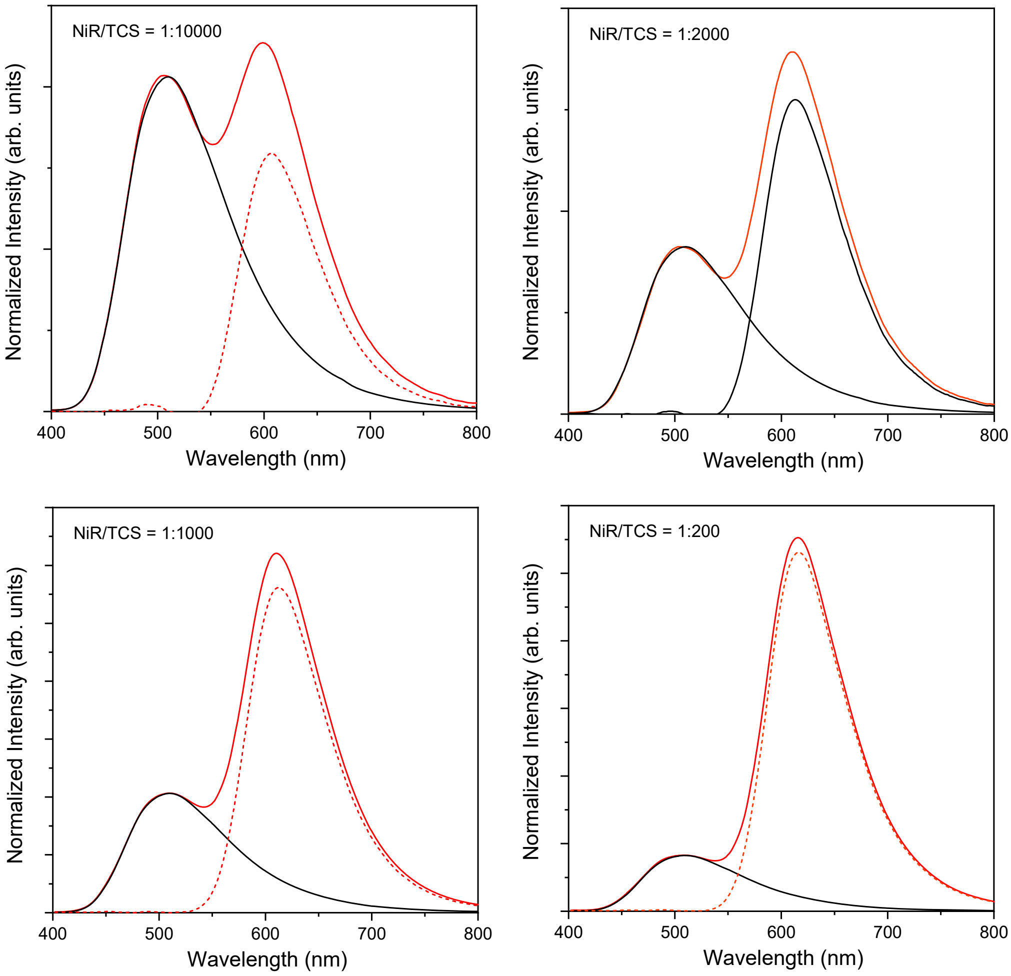

Supplementary Fig. 20 Fluorescence spectra of PTCS-NiR with variable ratios of NiR to TCS unit (red solid curve). Black solid curve represents the fluorescence spectrum of PTCS, which was normalized according to the intensity at 510 nm of the red solid curve. Red dashed curve indicates the absolute emission arising from NiR, which was obtained by subtracting the intensity of PTCS (black solid curve) from the spectra of PTCS-NiR (red solid curve).

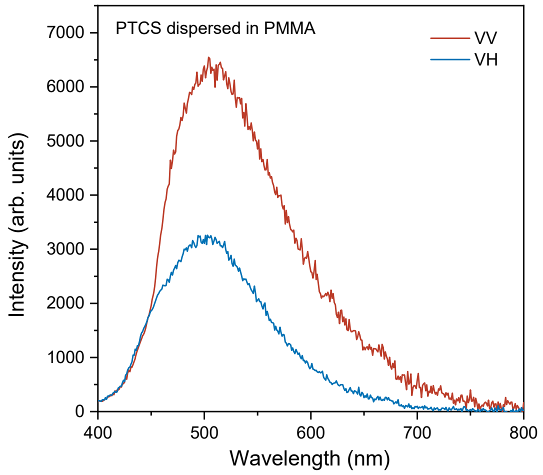

Supplementary Fig. 21 Fluorescence anisotropy spectra obtained from PTCS dispersed in polymethyl methacrylate (PMMA), where VV denotes vertical excitation and vertical emission, while VH denotes vertical excitation and horizontal emission.

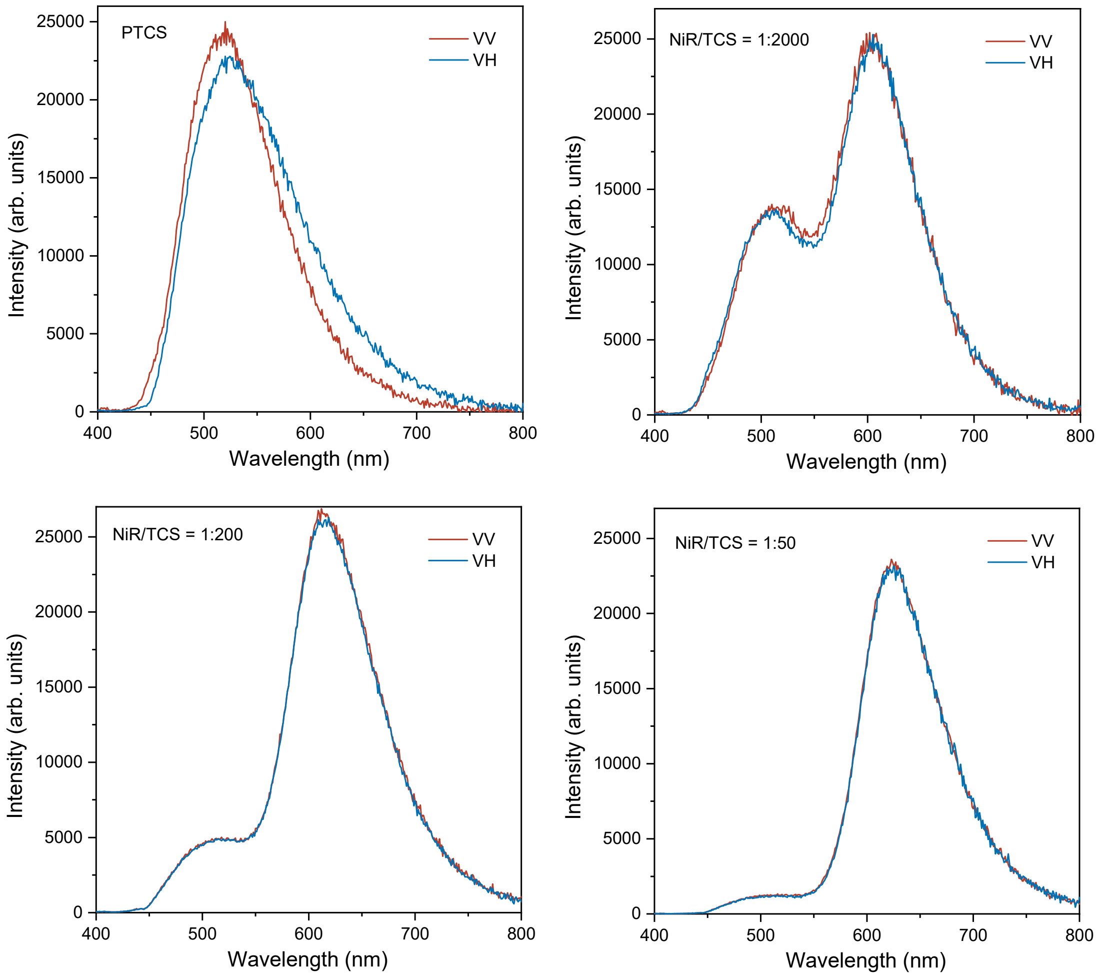

Supplementary Fig. 22 Fluorescence anisotropy spectra obtained from PTCS-NiR with different ratios of NiR to TCS unit, where VV denotes vertical excitation and vertical emission, while VH denotes vertical excitation and horizontal emission.

The anisotropy value is expressed as $(I_{VV} - I_{VH})/(I_{VV} + 2I_{VH})$. Actually, when ET occurs from TCS to NiR, there is an expected decrease in anisotropy. Therefore, the comparable ratios of I_{VV} and I_{VH} arising from the emission of donor TCS and acceptor NiR suggest efficient exciton energy migration within columnar TCS arrays.

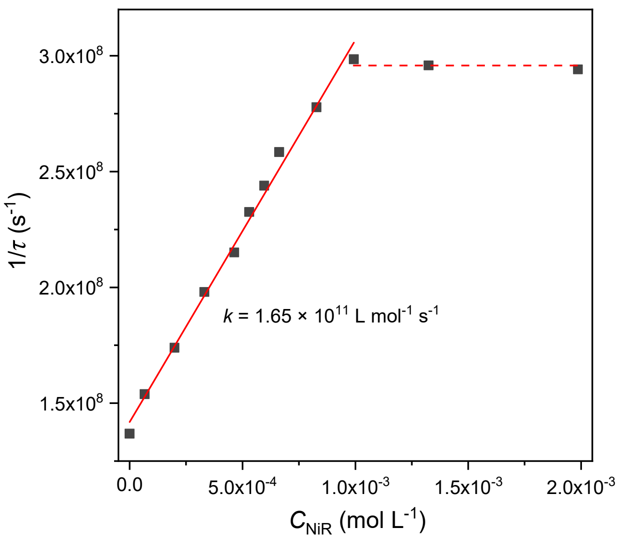

Supplementary Fig. 23 Plots of the reciprocal of the lifetime of the PTCS containing different concentrations of NiR against the concentrations of the NiR. The slope gives the corresponding second-order rate constant (k) for the exciton migration towards the acceptor. The concentration (C_{NiR}) was estimated by using the lattice parameters of PTCS determined by X-ray scattering.

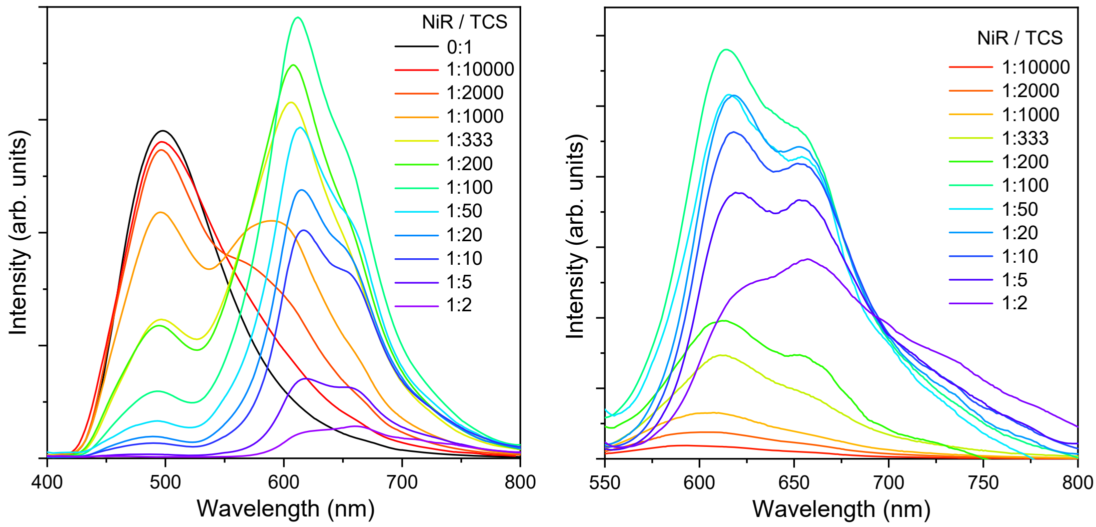

Supplementary Fig. 24 Fluorescence emission spectra of MTCS-NiR at 20 °C of the crystalline columnar phase with variable ratios of NiR to TCS unit. The excitation wavelength is 377 nm of the maximum absorption of MTCS (left) or 530 nm of the maximum absorption of NiR (right).

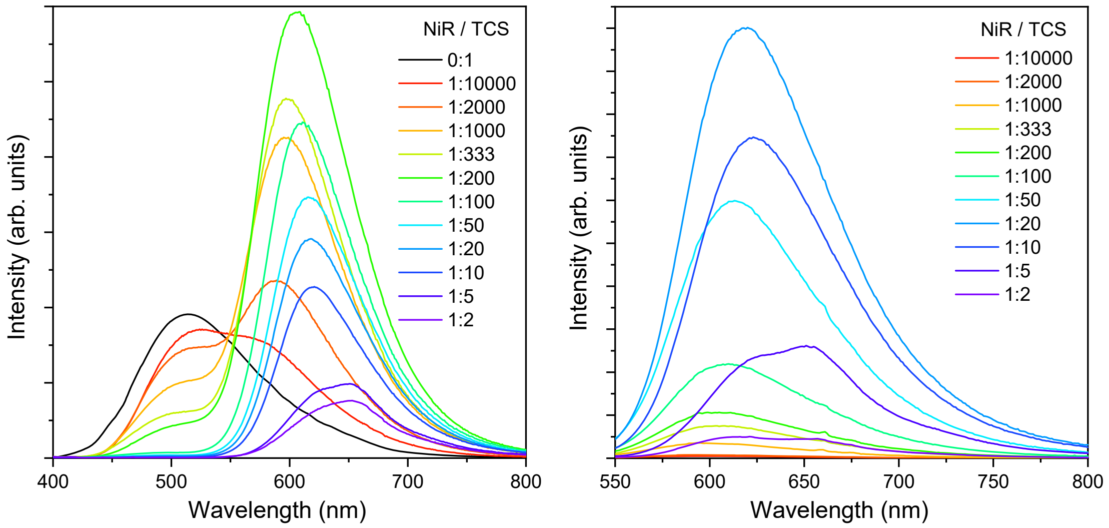

Supplementary Fig. 25 Fluorescence emission spectra of MTCS-NiR at 90 °C of the Col_h LC phase with variable ratios of NiR to TCS unit. The excitation wavelength is 377 nm of the maximum absorption of MTCS (left) or 530 nm of the maximum absorption of NiR (right).

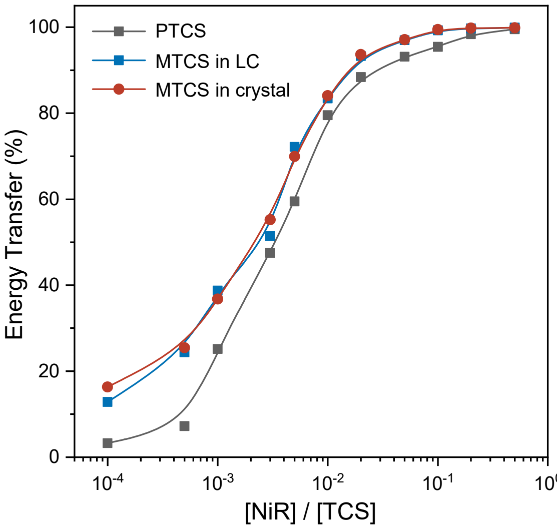

Supplementary Fig. 26 The ET efficiency as a function of the ratio of NiR to TCS unit of PTCS-NiR and MTCS-NiR in Col_h LC or crystalline columnar states.

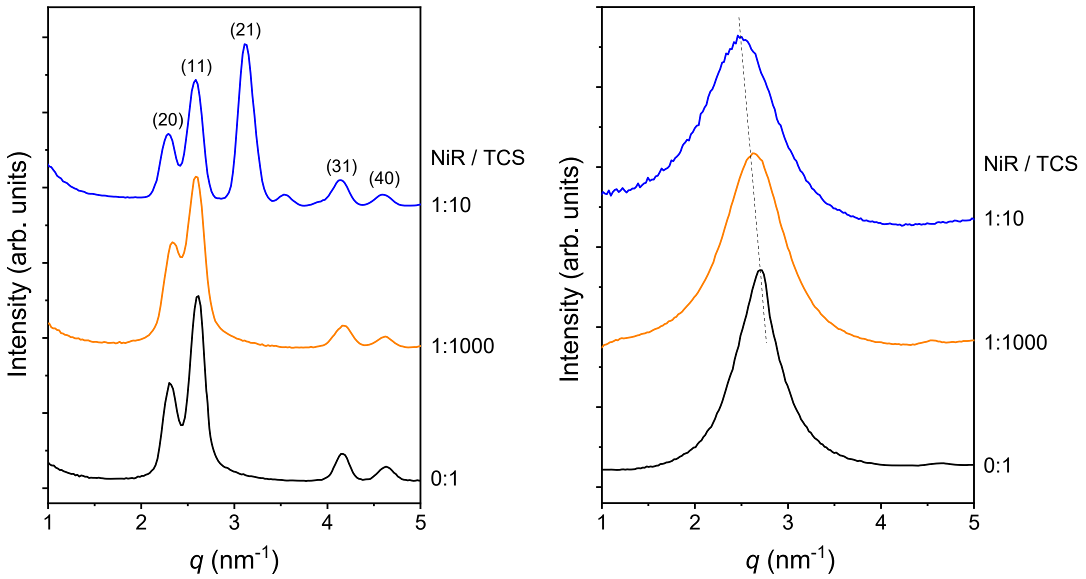

Supplementary Fig. 27 Representative X-ray scattering profiles of MTCS-NiR at 20 °C of the crystalline columnar phases (left) or at 90 °C of the Col_h LC phases (right) with different ratios of NiR to TCS unit. After intercalation of NiR, both the crystalline columnar and Col_h LC phases can be largely maintained, suggesting the co-stacking of NiR and MTCS into homogeneous columnar structures.

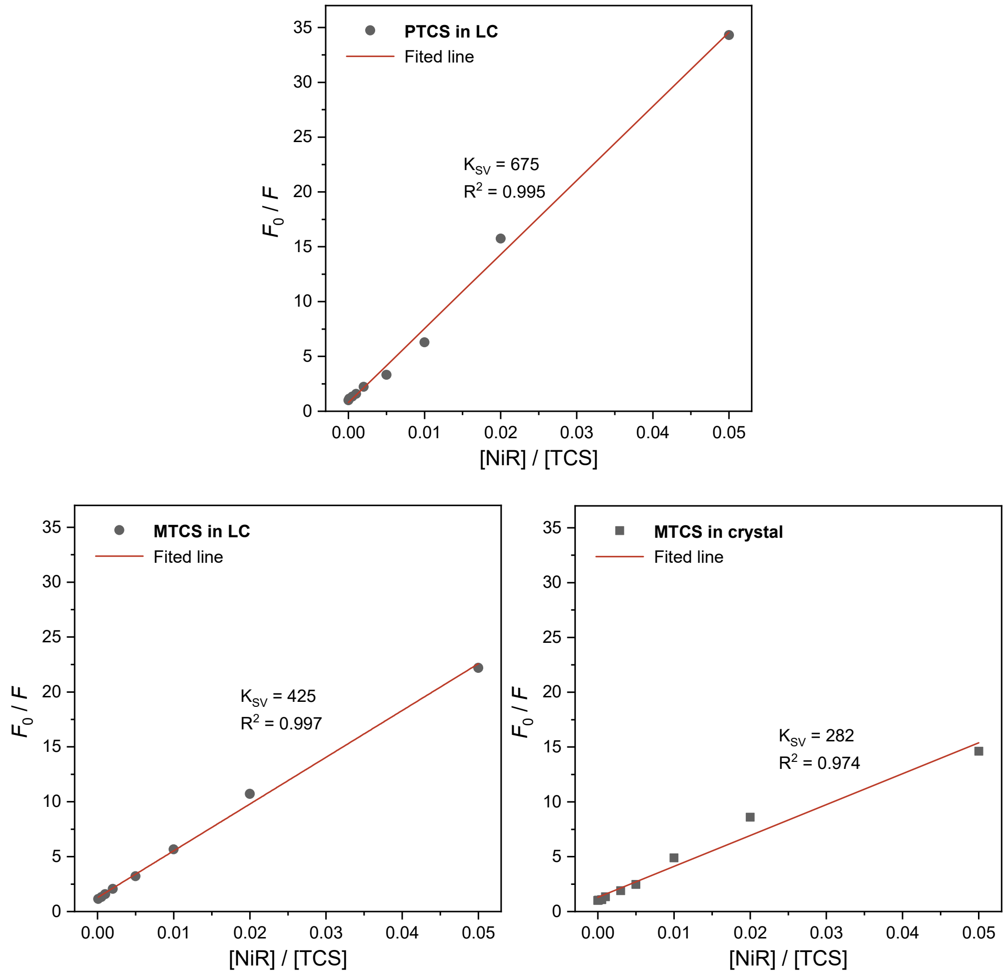

Supplementary Fig. 28 The linear fittings of K_{sv} of PTCS-NiR and MTCS-NiR in Col_h LC or crystalline columnar states versus the ratio of NiR to TCS unit.

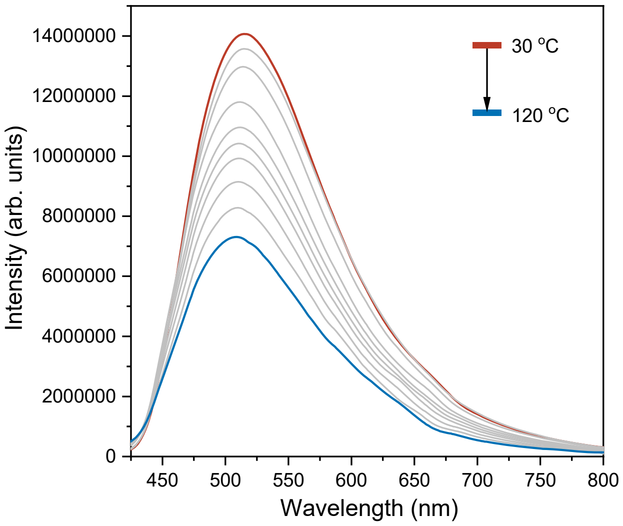

Supplementary Fig. 29 Temperature dependent fluorescence spectra of PTCS in the solid state from 30 to 120 °C with an interval of 10 °C. The excitation wavelength is 365 nm.

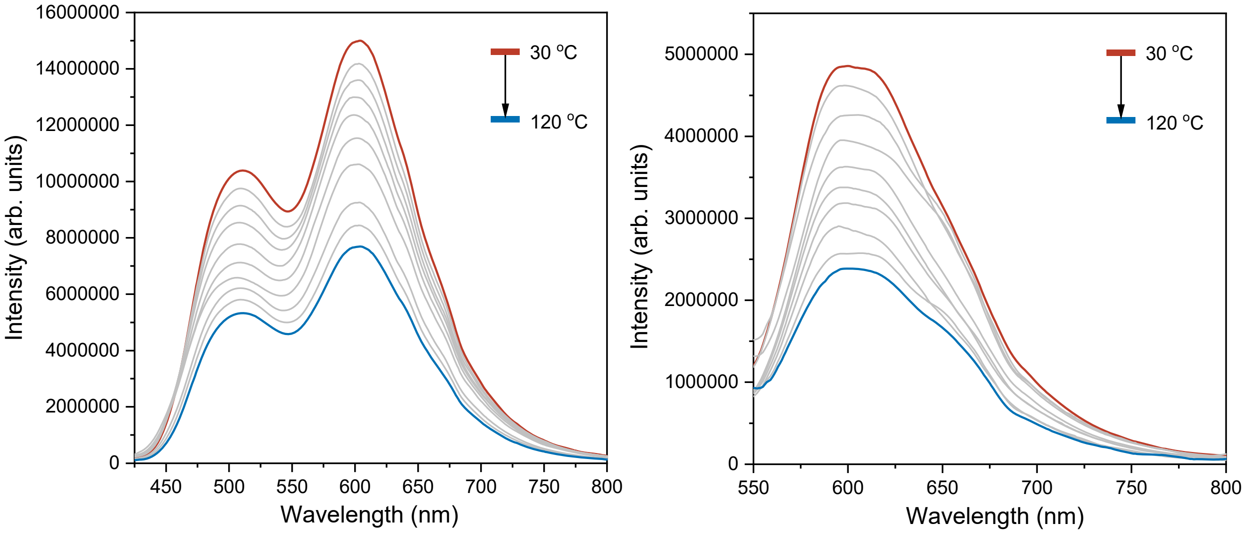

Supplementary Fig. 30 Temperature dependent fluorescence spectra of PTCS-NiR with the NiR/TCS ratio of 1:2000 in the solid state from 30 to 120 °C with an interval of 10 °C. The excitation wavelength is 365 nm (left) or 530 nm (right).

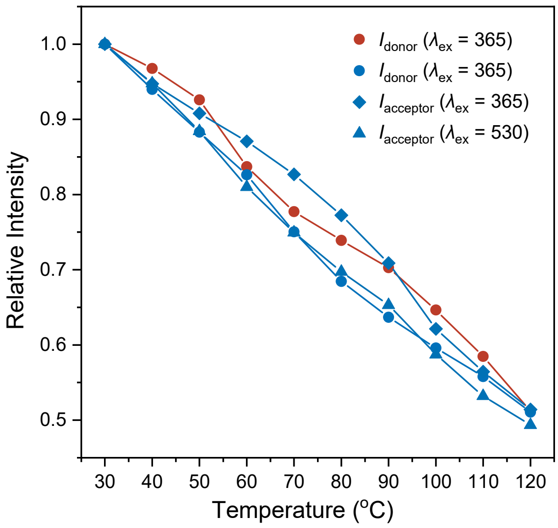

Supplementary Fig. 31 Relative emission intensity as a function of temperature. Red symbols and lines represent neat PTCS, and blue symbols and lines represent PTCS in the presence of the acceptor with NiR/TCS ratio of 1:2000. I_{donor}, the intensity arising from the donor; $I_{acceptor}$, the intensity arising from the acceptor. λ_{ex}, the excitation wavelength at 365 nm or 530 nm.

The emission intensity decreased with increasing temperature due to thermo-induced non-radiative decay. The decreasing trend appeared to follow a linear relationship throughout the entire system, suggesting stable light-harvesting performance with nearly constant ET efficiency and AE, regardless of temperature.

4. Supplementary Note 3: Control of ET via Photoresponsiveness

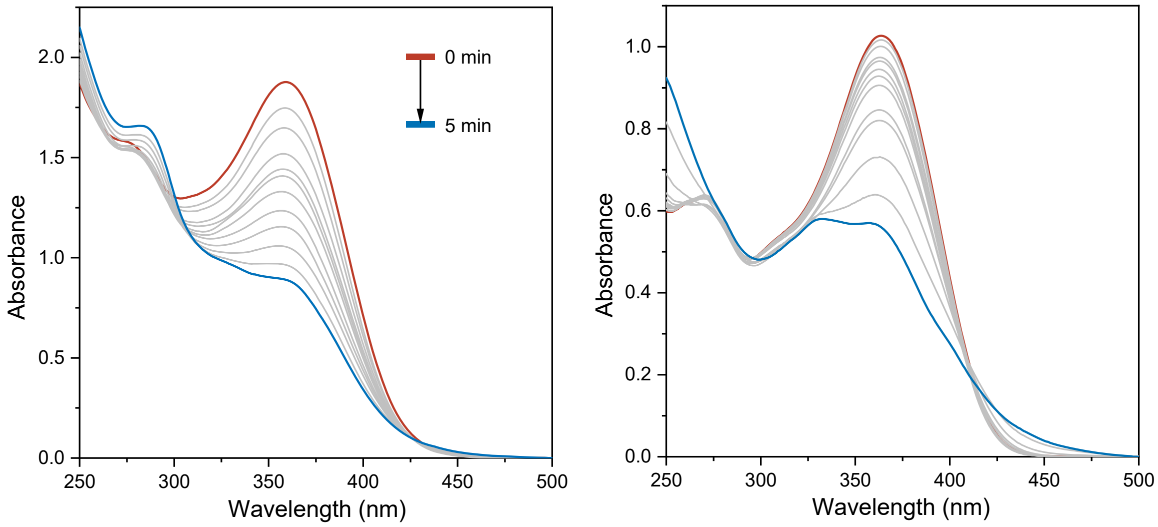

Supplementary Fig. 32 UV-vis absorption spectra evolution of PTCS (left) and MTCS (right) in dichloromethane solution during UV irradiation, showing similar *Z-E* isomerization process.

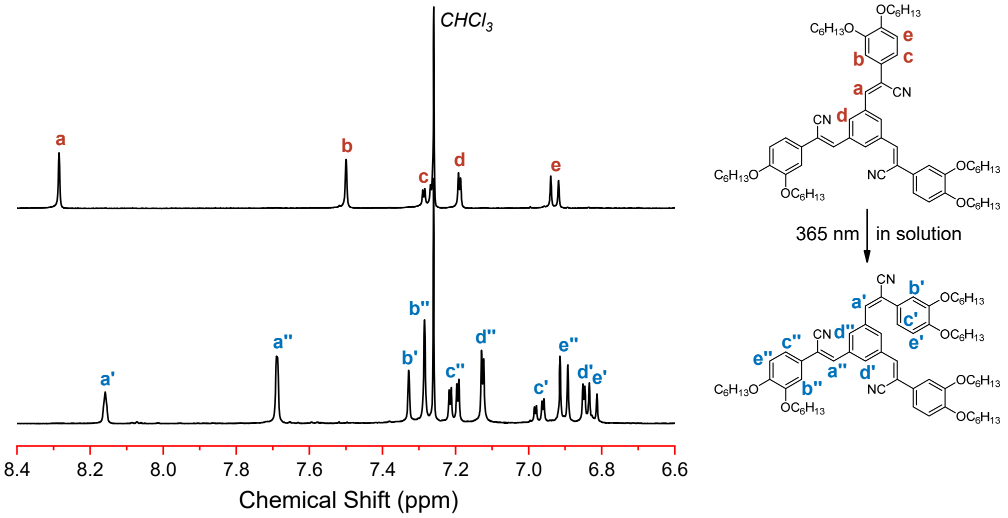

Supplementary Fig. 33 ^{1}H NMR spectra comparison for illustrating the photo-isomerization in solution from *Z,Z,Z*-MTCS to *Z,Z,E*-MTCS. The isomer *Z,Z,E*-MTCS was obtained by purification after UV irradiation of *Z,Z,Z*-MTCS in solution.

We used MTCS as the control compound to illustrate the photochemistry process because polymers are challenging to purify. A comparison of the ^{1}H NMR spectra of purified MTCS before and after UV irradiation clearly indicated that *Z-E* isomerization occurred for one of the three cyanostyrene arms in solution.

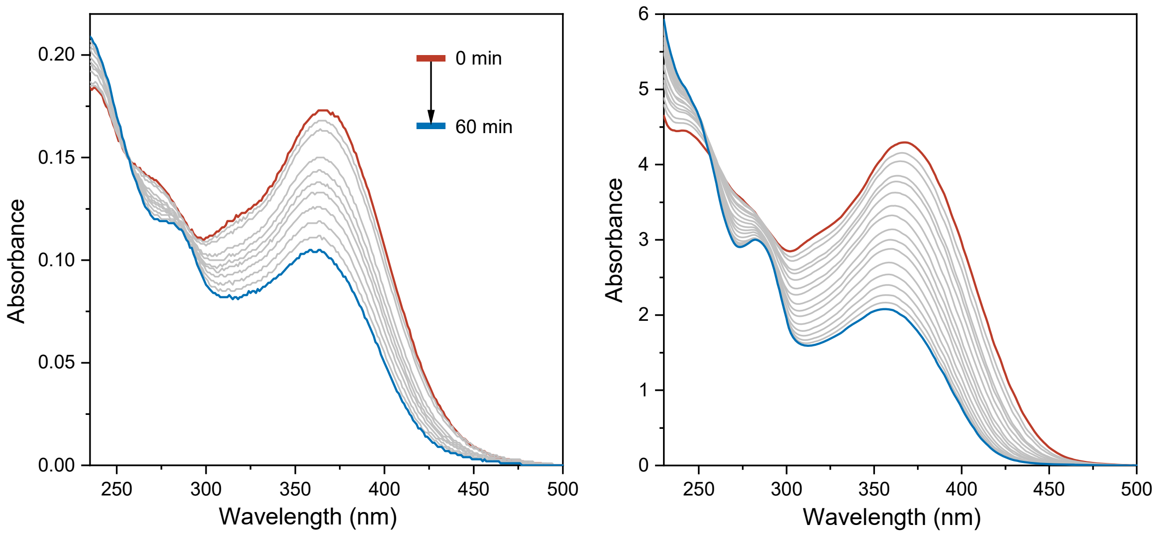

Supplementary Fig. 34 UV-vis absorption spectra evolution of PTCS (left) and MTCS (right) in the solid state during UV irradiation, indicating similar process of [2 + 2] cycloaddition.

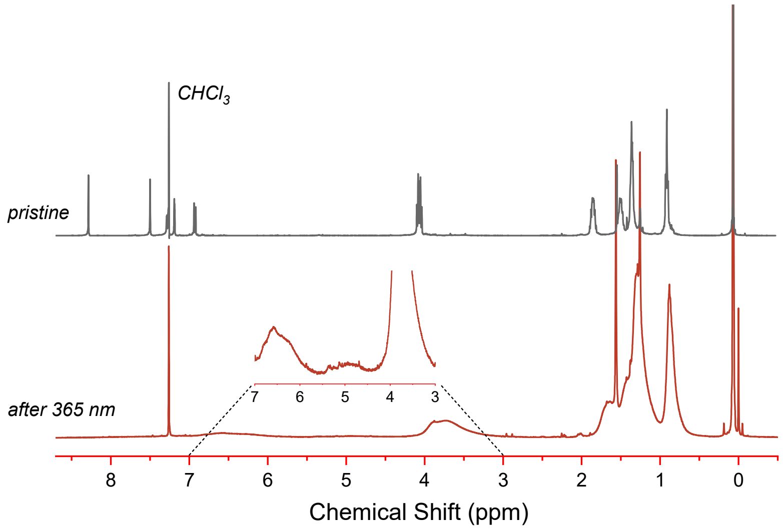

Supplementary Fig. 35 ^1H NMR spectra comparison of MTCS before and after UV irradiation in the solid state for illustrating the [2 + 2] cycloaddition between cyanostyrene arms. The red line after UV irradiation was obtained by proper irradiation to avoid excessive crosslinking and then removal of the residual MTCS through column chromatography.

PTCS did not dissolve in common organic solvents after UV irradiation, indicating the possible formation of crosslinked networks due to the [2 + 2] cycloaddition of the pendant TCS units in the solid state. To verify this assumption, we revisited the control compound MTCS. In this case, [2 + 2] cycloaddition might not lead to complete crosslinking, allowing for chemical structure identification. As expected, the ^1H NMR spectrum of MTCS after UV irradiation exhibited characteristic features of

polymer formation. This phenomenon was attributed to the intermolecular [2 + 2] cycloaddition of the cyanostyrene arms within the assembled supramolecular columns. Specifically, the peak around 5 ppm corresponded to the proton signal of cyclobutane generated by intermolecular [2 + 2] cycloaddition[3]. However, prolonged UV irradiation led to crosslinked networks even in MTCS due to its three photoactive cyanostyrene arms. Therefore, the insolubility of PTCS after UV irradiation can be rationalized by the intra-/inter-chain [2 + 2] cycloaddition of the pendent TCS units.

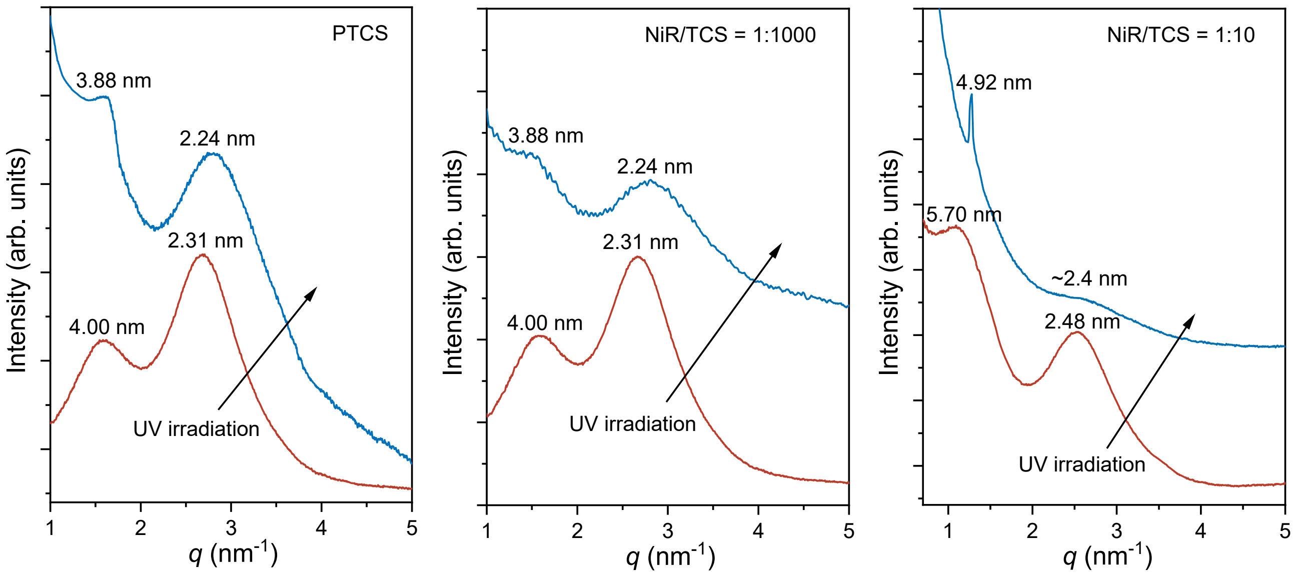

Supplementary Fig. 36 Representative X-ray scattering profiles of PTCS-NiR before and after UV irradiation, indicated with the corresponding *d* spacing of the scattering peak.

The structure variation upon UV irradiation was studied through X-ray scattering experiments. The photoactive unit TCS located at the core of the supramolecular column and surrounded by alkyl shells, indicates that light-induced [2 + 2] cycloaddition predominantly occurs within the column. As a result of intracolumnar crosslinking, the columnar dimension may shrink, causing a shift in the corresponding scattering peaks towards higher *q*-values. The hexagonal symmetry between columns can be maintained, evidenced by a consistent *d*-ratio of 1:1/√3 for both neat PTCS and PTCS-NiR at low NiR/TCS ratios. However, at high ratios, the two-dimensional hexagonal symmetry is disrupted. Despite this interruption, intracolumnar crosslinking under UV irradiation leads to noticeable columnar shrinking, resulting in decreased *d* values for the corresponding scattering signals.

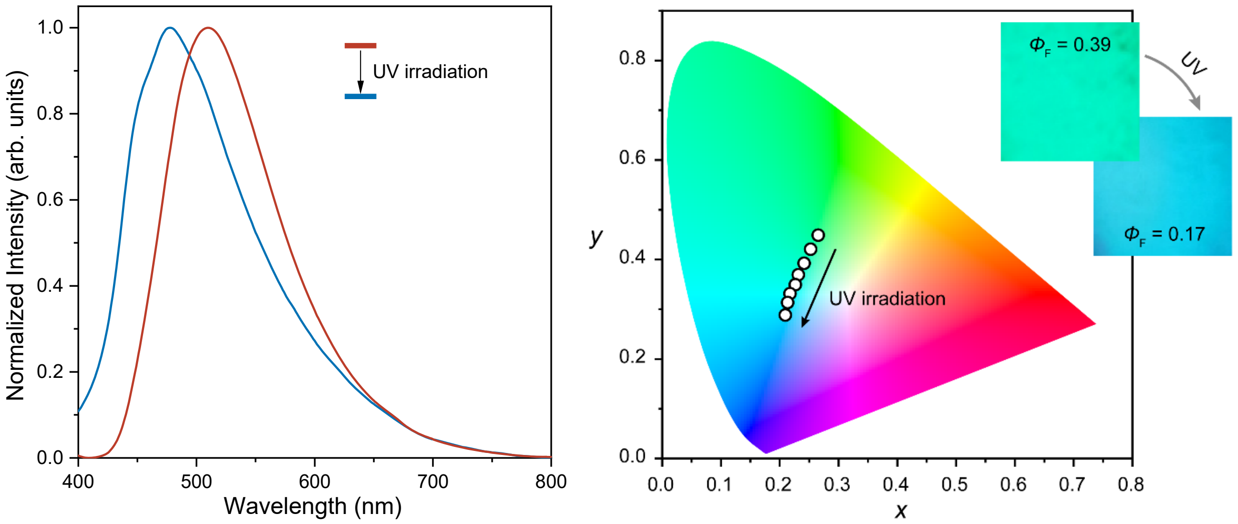

Supplementary Fig. 37 Normalized emission spectra of PTCS before and after UV irradiation in the solid state, and CIE chromaticity diagram showing the fluorescence color evolution of PTCS under UV irradiation. Insets showing the corresponding fluorescence images under UV illumination.

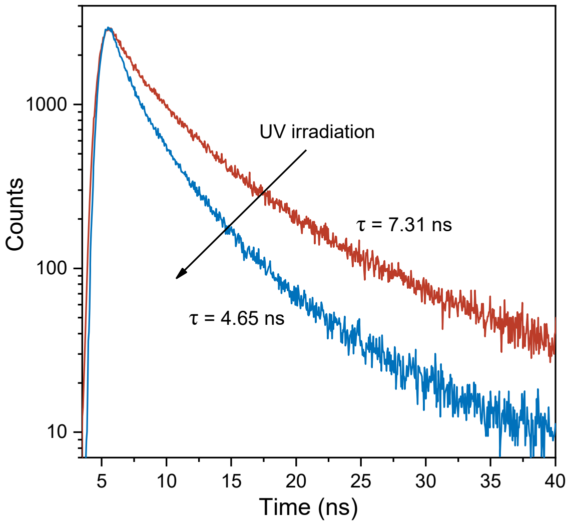

Supplementary Fig. 38 Fluorescence decay profiles of PTCS before and after UV irradiation in the solid state.

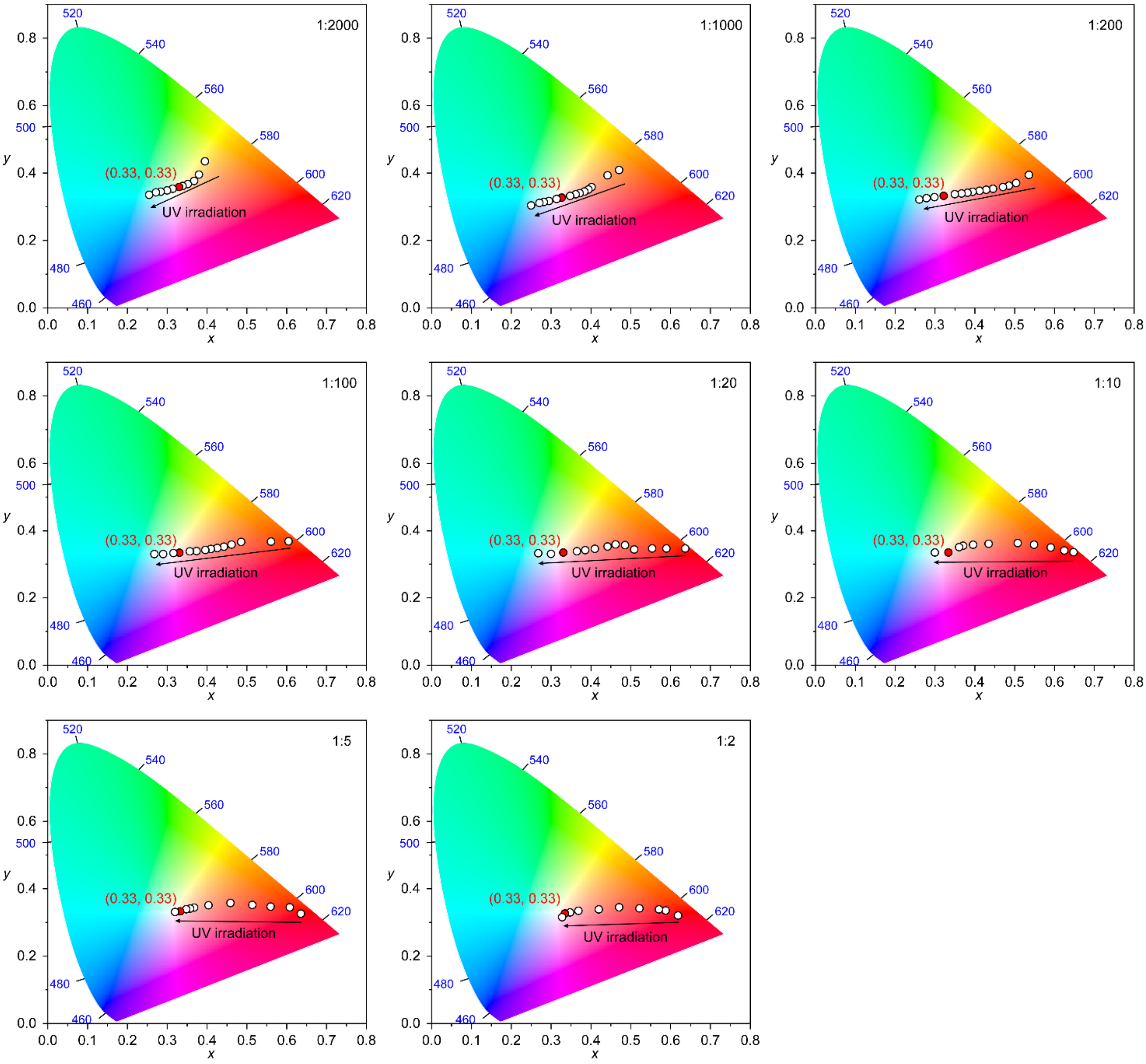

Supplementary Fig. 39 CIE chromaticity diagrams showing the fluorescence color evolution of PTCS-NiR with different ratios of NiR to TCS unit under UV irradiation. The arrows indicate the changing direction with irradiation duration. The color coordinate (0.33, 0.33) of white light emission is highlighted with red solid circles.

5. Supplementary References

1. Newkome, G. R. et al. Hexagonal terpyridine-ruthenium and -iron macrocyclic complexes by stepwise and self-assembly procedures. *Chem. Eur. J.* **8**, 2946-2954 (2002).
2. Das, A. & Theato, P. Multifaceted synthetic route to functional polyacrylates by transesterification of poly(pentafluorophenyl acrylates). *Macromolecules* **48**, 8695-8707 (2015).
3. Mu, B. et al. Positional isomerism-mediated copolymerization realizing the continuous luminescence color-tuning of liquid-crystalline polymers. *Macromolecules* **55**, 5332-5341 (2022).
